# Diagnosis and treatment complications of chronic otitis media

**DOI:** 10.1007/s00405-013-2506-0

**Published:** 2013-06-26

**Authors:** Jerzy Kuczkowski, Wojciech Sierszeń, Tomasz Przewoźny

**Affiliations:** Department of Otolaryngology, Medical University of Gdańsk, Smoluchowskiego 14, 80-214 Gdańsk, Poland

**Keywords:** Chronic otitis media, Complications

**Fig. 1:**
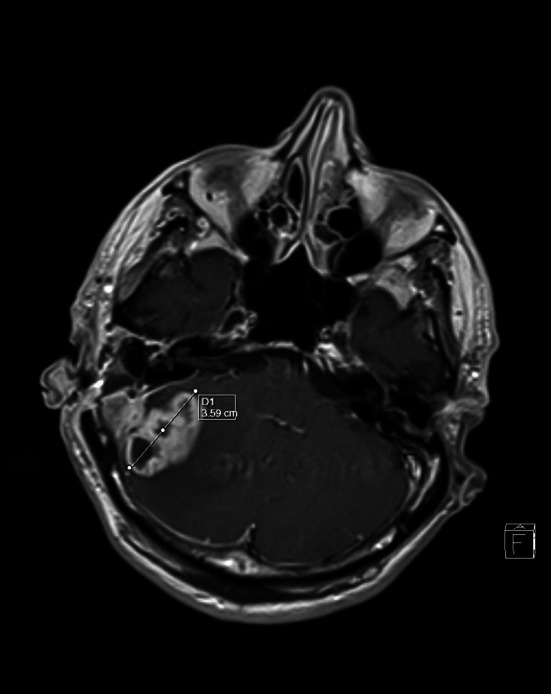
A 56-year-old patient with right-sided chronic otitis media with cholesteatoma complicated with a cerebellar abscessus. Left ear after radical mastoidectomy (MRI)

To the Editor,

The article by E. Yorgancilar et al. [1] presents interesting and very important clinical observations. The authors described clinical data and therapeutic approach to 121 patients with EC and IC complications of CSOM. They presented methods of treatment and epidemiological data of these complications (the most common was subperiosteal abscess—28.3 % and lateral sinus thrombophlebitis—19.5 %). The decrease in mortality in the authors’ clinic from 16.1 to 0 % is an excellent result and we would like to congratulate on it. Let us present some comments on this problem on the basis of our clinical experience. In the last years the outcome of treatment of these conditions is significantly better than in the past. Presently intracranial complications of CSOM are very rare in our Department as most commonly EC intricacy is observed. In the data (in the years 2001–11) of our Department we found 98 cases (86-EC and 12-IC) of CSOM otogenic complications. The most frequent EC intricacy was labyrinthitis and the most popular IC one was lateral sinus thrombophlebitis. Nowadays the symptoms of complications are less prominent than in the past due to antibiotic treatment of COM prior to hospital admission. The time required for full diagnosis of patients with complicated COM is reduced due to CT with contrast and MRI availability (useful to diagnose extradural abscess, subdural empyema, meningitis or thrombophlebitis of the dural veins). This technique is particularly useful in children [2] (Fig. 1).

In connection with the article let us ask some questions to the authors: Were all of the patients operated on primarily? Why were the extradural abscesses and the temporal lobe abscesses not evacuated during the mastoid surgery? Temporal lobe abscesses may be evacuated during ear surgery (by puncture and aspiration). In this method of temporal lobe abscesses evacuation neuronavigation is a very serviceable tool. Neurosurgical approach to the cerebellar abscess and to the abscess located in occipital area is preferred. In patients with LST treatment consists of canal wall down mastoidectomy, delamination of dura and puncture of sigmoid sinus. When perisinus abscess is absent and a patient does not present symptoms of sepsis the clot is not removed from sigmoid sinus [3]. In presence of perisinus abscess and signs of septicemia the sinus is punctured, the clot is removed and the jugular vein is ligated. We agree with the authors that there is no need to bind up the jugular vein in the case of LST without septicemia.
